# Supplementary material for: The epidemiology of gestational diabetes, gestation hypertension, and anemia in North Palestine from 2018 to 2020: A retrospective study
Source: PLoS One. 2024 Mar 29;19(3):e0301145. doi: 10.1371/journal.pone.0301145 (PMC10980252; doi:10.1371/journal.pone.0301145)
Supplement: Attachment — Submitted filename: Plos one review.docx [file pone.0301145.s002.docx]

**Introduction**

1. Well written
2. Please emphasize the novelty of the study and provide two or more related works in the context of Palestine.

**Methods**

1. Data exclusion criteria explained well.
2. Please provide a detailed explanation for why we did not consider different available methods for imputing missing values in electronic health data or health data records, given that missing data in your final samples is a significant concern.
3. Extend Data analysis a bit more, with more specification.

**Results-** Well written

**Discussion** –

1. Well written.
2. Please make it a bit short in length and focus on your objective and future implication in terms of Palestine.

**Conclusion** – well written
